# Determinants of COVID-19 Vaccine Acceptance among Dental Professionals: A Multi-Country Survey

**DOI:** 10.3390/vaccines10101614

**Published:** 2022-09-26

**Authors:** Mohammad Zakaria Nassani, Mohammed Noushad, Samer Rastam, Mudassir Hussain, Anas B. Alsalhani, Inas Shakeeb Al-Saqqaf, Faisal Mehsen Alali, Amir Mohiddin Demachkia, Renata Marques de Melo, Mohammed Arshad, Norhayati Luddin, Adam Husein, Zeeshan Qamar, Pradeep Koppolu, Mahmoud Darwish, Ahmad Salim Abdalla Nassar, Adnan Habib, Firas Suleyman, H. M. Khuthija Khanam, Salah A. Yousief, Sadeq Ali Al-Maweri, Nafeesa Tabassum, Abdulaziz Samran, Mohiddin R. Dimashkieh, Mohammed Sadeg Al-Awar, Bassel Tarakji

**Affiliations:** 1 Department of Restorative and Prosthetic Dental Sciences, College of Dentistry, Dar Al Uloom University, Riyadh 13313, Saudi Arabia; 2 Department of Clinical Sciences, Vision College of Medicine, Vision Colleges, Riyadh 11691, Saudi Arabia; 3 Department of Community and Preventive Dentistry, Karachi Medical and Dental College, Karachi 74700, Pakistan; 4 Department of Oral Medicine and Diagnostic Sciences, Vision College of Dentistry and Nursing, Vision Colleges, Riyadh 11691, Saudi Arabia; 5 School of Social Sciences, Universiti Sains Malaysia, George Town 11800, Malaysia; 6 Department of Oral and Maxillofacial Surgery and Diagnostic Sciences, College of Dentistry Prince Sattam Bin Abdulaziz University, Al-Kharj 16278, Saudi Arabia; 7 Department of Dental Materials and Prosthodontics, São Paulo State University (UNESP), São José dos Campos 01049-010, Brazil; 8 Department of Public Health Dentistry, Kannur Dental College, Kannur 670612, India; 9 Prosthodontic Unit, School of Dental Sciences, Universiti Sains Malaysia, Kubang Kerian 16150, Malaysia; 10 Department of Preventive and Restorative Dentistry, College of Dental Medicine, University of Sharjah, Sharjah 27272, United Arab Emirates; 11 Department of OMFS and Diagnostic Sciences, Riyadh Elm University, Riyadh 13244, Saudi Arabia; 12 Department of Preventive Dental Sciences, College of Dentistry, Dar Al Uloom University, Riyadh 13313, Saudi Arabia; 13 Department of Prosthetic Dental Sciences, Vision College of Dentistry and Nursing, Vision Colleges, Riyadh 11691, Saudi Arabia; 14 Prosthodontics Department, Faculty of Dentistry, Suez Canal University, Ismailia Governorate 8366004, Egypt; 15 Ministry of Health, Amman 11118, Jordan; 16 Department of Conservative Dentistry, College of Dentistry, Aleppo University, Aleppo 12212, Syria; 17 Department of Prosthodontics, Hamidiye Faculty of Dentistry, University of Health Sciences, Istanbul 34668, Turkey; 18 Crown and Bridge Department, Faculty of Oral and Dental Medicine, Al Azhar University, Assuit Branch 71524, Egypt; 19 College of Dental Medicine, QU Health, Qatar University, Doha P.O. Box 2713, Qatar; 20 Department of Diagnostic Sciences, College of Dentistry, Dar Al Uloom University, Riyadh 13314, Saudi Arabia; 21 Faculty of Applied Science, Amran University, Amran 363655, Yemen; 22 Department of Medical Laboratory, College of Medical Science, Al-Razi University, Sana’a 1152, Yemen

**Keywords:** COVID-19, vaccine acceptance, dentists, dental students, low-income, high-income

## Abstract

Purpose: This study sought to investigate the acceptance rate and associated factors of COVID-19 vaccines among dentists and dental students in seven countries. Material and Methods: A structured questionnaire prepared and guided by the report of the SAGE Working Group on Vaccine Hesitancy was distributed among groups of dentists and dental students in seven countries across four continents. Results: A total of 1527 subjects (850 dentists and 677 dental students) participated in this survey. Although 72.5% of the respondents reported their intention to accept COVID-19 vaccines (dentists: 74.4%, dental students: 70.2%), there was a significant difference in agreement between dentists/dental students across countries; generally, respondents in upper-middle-, and high-income countries (UM-HICs) showed significantly higher acceptance rates compared to those in low- and lower-middle income countries (L-LMICs). Potential predictors of higher vaccine acceptance included being a dentist, being free of comorbidity, being well-informed about COVID-19 vaccines, having better knowledge about COVID-19 complications, having anxiety about COVID-19 infection, having no concerns about the side effects of the produced vaccines and being a resident of an UM-HIC. Conclusion: The results of our survey indicate a relatively good acceptance rate of COVID-19 among the surveyed dentists and dental students. However, dentists and dental students in L-LMICs showed significantly lower vaccine acceptance rates and trust in COVID-19 vaccines compared to their counterparts in UM-HICs. Our results provide important information to policymakers, highlighting the need for implementation of country-specific vaccine promotion strategies, with special focus on L-LMICs.

## 1. Introduction

The COVID-19 pandemic, caused by SARS-CoV-2, has been one of the most devastating and lethal public health emergencies the world has witnessed during the past century. Due to the nature of their work, healthcare workers (HCWs) have been one of the most vulnerable groups, not only to getting infected with the virus, but also to transmitting it to their patients, colleagues, and relatives. Among HCWs, dentists are at increased risk of being infected, owing to their close proximity to their patients’ virus transmission routes during practice, and routine exposure to aerosol emissions and spatters that may be contaminated with high viral loads [1]. Although the World Health Organization (WHO) has issued statements specific to aerosol-generating procedures and the need for the use of enhanced personal protective equipment (PPE), access to these is very limited in several countries, mainly in low-income countries (LICs) [2].

Currently, the most trusted pathway to combat the COVID-19 pandemic is through achieving the optimum immunization necessary for population immunity. This can only be achieved by effective implementation of large-scale vaccination strategies globally. Although HCWs, including dentists, have been prioritized globally to receive vaccination against COVID-19 in the first phase of the rollout, vaccine hesitancy and the extreme disparity in access to vaccines across regions and economic groupings could prove to be major hurdles in attaining population immunity [3].

Even though there have been numerous studies on COVID-19 vaccine hesitancy among HCWs, studies among dentists and dental students are limited. One of the first studies on COVID-19 vaccine acceptance among dentists indicated an acceptance rate of 82% [4]. Another similar study indicated a higher rate of vaccine acceptance among dentists as compared to dental students [5]. A recent scoping review also indicated similar findings, suggesting a worldwide COVID-19 vaccine refusal rate of 19% among practicing dentists and 24.9% among dentistry college students. Major reasons for COVID-19 refusal included concerns about the safety, efficacy, side effects, or expedited development of COVID-19 vaccines, and lack of trust in vaccines and vaccination, healthcare organizations, government, pharmaceutical companies, etc. [6]. Risk awareness and safety perception among dental professionals are other possible factors that could affect vaccine acceptance. Dentists have been shown to have an acceptable level of risk awareness [7]. A study correlating safety perception with vaccine acceptance among dental health care workers revealed that dentists felt less safe with regular SARS-CoV-2 testing and had higher vaccine acceptance, when compared with dental assistants and hygienists [8].

Although most high-income countries (HICs) have vaccinated most of their HCWs, including dentists, LICs are still lagging behind [9]. Since comparative studies of vaccine acceptance among dentists and dental students in low- and high-income countries are limited, our study aimed to fill this gap. Hence, this study sought to investigate the acceptance rate and associated factors of COVID-19 vaccines among dentists and dental students in seven countries across four continents, based on the income index.

## 2. Material and Methods

### 2.1. Study Design, Participants, and Ethical Approval

This online questionnaire-based study is a part of a large multinational survey conducted among various healthcare professionals [10]. The present study was conducted among dentists and dental students in seven countries, namely Pakistan, Egypt, India, Saudi Arabia, Malaysia, Brazil, and Turkey from February to the middle of April 2021. The countries were categorized into two groups based on the income index: three low- and lower-middle-income countries (L-LMICs) and four upper-middle income and high-income countries (UM-HICs).

All practicing dentists and senior dental students (clinical dental students) were eligible to participate. However, dental students in the preclinical level were not eligible to participate. The protocol of this study was approved by the research committee of the College of Dentistry, Dar Al Uloom University, Saudi Arabia (COD/IRB/2020/2). Participation was voluntary and the participants provided informed consent on the survey platform before proceeding to the survey items. The participants’ anonymity was guaranteed during the data collection process.

### 2.2. Survey Methods

An online questionnaire was used to collect data from the participants. A detailed description of the questionnaire items is already mentioned in a previous study [10]. The report of the SAGE Working Group on Vaccine Hesitancy and opinions from experts in the field were used in preparing the questionnaire [11]. The questionnaire consisted of 23 closed-ended questions divided into four sections: (1) socio-demographic and professional data (age, gender, nationality, profession, and place of work/study, i.e., governmental/private/both), (2) dentists’ general attitudes toward vaccines (six items), (3) respondents’ trust in health care system in the context of COVID-19 vaccines (two items), and (4) acceptance of COVID-19 vaccine (seven items).

We used six items to measure general attitudes toward the vaccine on a five-point Likert scale. The participants were first asked if vaccines were really necessary to combat the pandemic. Perceived trust in vaccine manufacturers was assessed using questions that included participants’ trust in vaccines of only specific companies, following of recommended development and production guidelines by vaccine manufacturers, commercial profiteering concerns, risk of side effects of vaccines, and manufacturers’ disclosure of the side effects of vaccines. We used two items to measure the attitudes of participants toward the health care system on a five-point Likert scale. We asked participants if they were happy with the health authorities’ management of the pandemic and vaccination campaigns. We then used seven items to measure participants’ vaccine intention on a six-point Likert scale. Participants were first asked if vaccines should be made mandatory and if they intended to get vaccinated. Further questions included fear of the vaccine, care for others who would be in greater need for the vaccine, intention to protect others with weaker immunity, willingness/unwillingness to take the vaccine if required to pay for it, and fear of side effects from a second dose.

Prior to distribution of the questionnaire, a pilot study was conducted among 10 dentists to ensure clarity and reliability of the questions. The questionnaire required less than five minutes to complete, and the participants were reminded only once, on failure to complete the survey form.

### 2.3. Exploratory Variables

The following potential exploratory factors were considered: socio-demographic factors such as gender, age, country of residence, work setting/study place; presence of pre-existing co-morbidity (any chronic medical condition such as diabetes mellitus, cardiovascular diseases, asthma/COPD, cancers, and/or renal disease, etc.); history of prior infection with COVID-19; participants’ level of knowledge of COVID-19 vaccine development; perception of COVID-19 severity; compliance with government COVID-19 guidelines; and anxiety toward contracting COVID-19.

### 2.4. Statistical Analysis

All statistical analyses were performed using IBM SPSS Statistics version 25.0 (IBM Corp. Released 2017. IBM SPSS Statistics for Windows, version 25.0. IBM Corp, Armonk, NY, USA). Descriptive data were presented as frequencies and percentages for different parameters. The chi-square statistic was used to assess the differences in COVID-19 vaccine acceptance across study subgroups/demographics. A logistic model was created with “vaccine acceptance” as the outcome variable and the exploratory variables as predictors. Odd ratios (ORs) and their 95% confidence intervals (CIs) were calculated. The significance level was set at *p* < 0.05.

## 3. Results

Overall, 1527 participants (850 dentists and 677 dental students) completed the survey. Of these, 767 were from three L-LMICs, and 760 from four UM-HICs. Demographics and characteristics of the study population are presented in Table 1. Table 2 and the Table S1 present proportions of dentists/dental students who agreed to the various survey questions based on income index. Overall, more than two-thirds of the participants (76.2%) recognized the importance of vaccines to overcome the COVID-19 pandemic and getting back to normal life, with clear variations across countries (ranging from 44.7% in Egypt to 98.1% in Malaysia). Concerning the potential side effects of vaccines, around half of the participants (47.5%) believe that COVID-19 vaccines may have some side effects, again with apparent differences across countries (ranging from 24.4–73.8%). With respect to dentists/dental students’ trust in the health authorities and their dealing with the COVID-19 pandemic, the results varied greatly across countries and ranged from as low as 5.5% (in Brazil) to as high as 92.4% (in Saudi Arabia), with an overall satisfaction score of 62.4%. The acceptance rate of the COVID-19 vaccine varied greatly across countries (33–97.5%), with an overall acceptance rate of 72.5%. Subjects in Brazil and Malaysia showed the highest acceptance rates (96.3% and 97.5%, respectively), while those from Egypt showed the lowest (33%) (Table 2, Table S1). Moreover, our results showed a significant association between country income and respondents’ trust in COVID-19 vaccines and their vaccine acceptance. Concerns about side effects from COVID-19 vaccines was significantly higher among dentists/dental students from L-LMICs (*p* < 0.001). On the other hand, vaccine acceptance was significantly higher among participants from UM-HICs (*p* < 0.001). No significant difference was identified among the two groups of countries with regard to trust in health authorities’ management of the pandemic (Table 2).

Table 3 presents COVID-19 vaccine acceptance among dentists/dental students according to country and demographics. It is noteworthy that age, sex, profession, comorbidity and previous COVID-19 infection had almost no association with vaccine acceptance among the study population across most of the surveyed countries. The two exceptions were India and Turkey where older participants and dentists showed higher levels of vaccine acceptance (*p* < 0.05). Among the overall study population, five factors were associated with greater acceptance of COVID-19 vaccine. These include updating self on the development of COVID-19 vaccines, higher perception of the severity of COVID-19, good compliance with COVID-19 preventive guidelines, higher anxiety level about contracting COVID-19 and lack of concern about the side effects of COVID-19 vaccines (Table 3).

When participating countries were grouped according to income, the bivariate analysis revealed significantly higher COVID-19 vaccine acceptance among dentists/dental students from UM-HICs (78.5% versus 66.6% for L-LMICs, *p* < 0.001). This trend of higher acceptance in UM-HICs was dominant among most demographics included in the survey (Table 4).

Logistic regression statistics identified eight predictors of vaccine acceptance among the surveyed population. Lower acceptance can be anticipated among older dentists/dental students, dental students, those with comorbidity, and those who had concerns about the side effects of the produced vaccines. On the contrary, higher COVID-19 acceptance can be foreseen among dentists/dental students who followed the updates about COVID-19 vaccines, those who perceived COVID-19 to be a severe disease, those with higher levels of anxiety about contracting COVID-19 and those who were residents of UM-HICs (Table 5).

## 4. Discussion

The role of dentists and dental students in the fight against COVID-19 is of utmost value [12,13], not only in terms of safe provision of oral/dental care during the pandemic, but also in educating and motivating the public to accept COVID-19 vaccines and obeying preventive measures. The current survey was carried out in this context to shed light on dentists and dental students’ acceptance of COVID-19 vaccines and related determinants of vaccine acceptance. Although the overall vaccine acceptance rate in our study was good, there was heterogeneity between the two groups of countries, with dentists and dental students in L-LMICs indicating significantly lower vaccine acceptance rates and trust in COVID-19 vaccines compared to their counterparts in UM-HICs.

Acceptance of COVID-19 vaccines among dentists and dental students has varied across countries. For example, a survey of Italian dentists revealed that a high percentage (82%) were willing to take a COVID-19 vaccine [4]. Similarly, 86% of the 529 dentists in a study in Lebanon expressed their willingness to get vaccinated against COVID-19 [14]. These figures are similar to the results of our study among participants in India and Turkey (81.2% and 84.9%, respectively). Chowdhury et al. [6] reported an overall refusal rate of COVID-19 vaccine, 19% being among 2983 dentists from 11 countries worldwide. They also found that the overall refusal rate of COVID-19 vaccines was 24.9% among 7805 dental students from 23 countries. These results are close to those identified in our study (overall refusal rate, 25.6% among dentists and 29.8% among dental students). In a survey of three dental schools in the United States of America, 44% of the dental students indicated hesitancy to take the COVID-19 vaccine and only 56% indicated their intention to take it once approved by the FDA [15]. Another global survey among dental students in 22 countries reported an overall hesitancy of 22.5% and rejection rate of 13.9% [16]. In a survey of Palestinian dental students, the acceptance rate of the vaccine was 57.8%, whereas 27% of the students were hesitant to take the vaccine and 14.9% showed no intention for vaccination [17]. Results of our survey among dentists and dental students in seven countries indicate a good COVID-19 vaccine acceptance rate of 72.5%. However, this figure can be considered less than optimal among the population of dentists/dental students considering that the risks of COVID-19 clearly exceeded the risks of vaccination for most individuals. Further research, perhaps of qualitative design, is recommended to understand the reasons behind this finding.

While an acceptance rate of 72.5% could be just enough to attain population immunity, it also reflects the dentists/dental students’ fair awareness of the need for vaccines to combat the pandemic. However, the wide heterogeneity in vaccine acceptance across the surveyed countries (33% in Egypt, 62.5% in Saudi Arabia, and 97.5% in Malaysia) and within each group, coupled with the participants’ beliefs regarding the potential side effects of COVID-19 vaccines (73.8% in Egypt, 45.6% in Saudi Arabia, and 24.4% in Brazil) stresses the need to adopt country/culture-specific strategies to educate the dental team on the importance of vaccines to attain global population immunity. Our results suggest concern about the potential side effects of COVID-19 vaccines among a considerable proportion of the study population (47.5%) indicating less than optimal trust in the outcomes of the available vaccines. This warrants implementation of further assurance strategies from the pertinent health authorities to promote confidence in the vaccines and motivate hesitant dentists/dental students to accept the vaccine. Fear of infection and the lethal threat of COVID-19 may have led to increased acceptance rates of COVID-19 vaccine among our study population.

A key finding in our study is the significantly lower vaccine acceptance rates and trust in COVID-19 vaccines among dentists and dental students in L-LMICs compared to their counterparts in UM-HICs (Table 4). This is similar to the results of a recent multi-country survey that reported greater vaccine hesitancy among dental students in 22 L-LMICs [16]. Interestingly, the finding of a lower vaccine acceptance among dentists/dental students from L-LMICs was apparent across the majority of the investigated demographics in this survey (Table 4). The perception of dentists/dental students in the L-LMICs of the various challenges/barriers for vaccine rollout and inequality in vaccine distribution/access to vaccine, in comparison with UM-HICS, coupled with uncertainty and lack of reliable sources about the spread of the disease and actual infection rates may explain such a negative finding [18].

Demographic factors such as older age and presence of systemic diseases have been shown to be associated with greater COVID-19 vaccine acceptance among the general population [19,20,21,22]. Results of our study among dentists and dental students however seem to be contrary to the above findings and warrant further investigations to identify the factors that are implicated in such findings. Nonetheless, it is interesting to note that awareness/personal COVID-19-related factors such as updating self on the development of COVID-19 vaccines, perception of the severity of COVID-19, compliance with COVID-19 preventive guidelines, anxiety about contracting COVID-19 and concern about the side effects of COVID-19 vaccines, were associated with greater vaccine acceptance. This emphasizes the need for healthcare planners to educate dentists and dental students on the development, safety profile and efficiency of the vaccines.

Amid the world’s largest vaccination drive, information regarding the determinants of vaccine hesitancy can be of much value for policymakers and healthcare providers. In other words, a thorough understanding of the factors that affect vaccine hesitancy can guide policymakers and healthcare providers to tailor strategies that reduce vaccine hesitancy and promote vaccine acceptance, and hence enhance the efficiency of vaccination campaigns to attain herd immunity. Our study has addressed eight potential predictors of vaccine acceptance among the surveyed dentists and dental students. Remarkably, when awareness about COVID-19 disease and its vaccines is present, acceptance of COVID-19 vaccines is expected. The expected lower hesitancy rates towards COVID-19 vaccines among dental students underlines the need to target them during vaccination campaigns to combat hesitancy among them. Unfortunately, our results suggest greater hesitancy among dentists and dental students in L-LMICs compared to their counterparts in UM-HICs, which is in line with the findings of a previous study among dental students [13]. As mentioned earlier, this could be the result of vaccine inequality and the hurdles in vaccine procurement and distribution in L-LMICs [23,24]. This could be a major barrier towards attaining population immunity in L-LMICs and calls for urgent action from the international community.

Despite the fact that our study is one of few studies that addressed COVID-19 vaccine hesitancy among dentists and dental students on an international level, a number of limitations can be identified. Firstly, our study provides information on vaccine acceptance at a specific point in time only. Since vaccine acceptance is time, place, and context dependent, individuals’ attitudes towards its acceptance are expected to change over time, necessitating further studies at different points in time. Such studies could provide useful information in potential future pandemics as well. Another limitation is the mode of recruitment of participants. Since we used the convenience sampling technique, our results may not represent dentists/dental students at large. However, they do provide valuable information for governments and public health experts. The web-based self-administration mode of survey that we used is another limitation which could be a source of bias. However, due to the pandemic related restrictions, this mode was the best available.

## 5. Conclusions

Results of our survey among dentists and dental students in seven countries indicate a good COVID-19 vaccine acceptance rate of 72.5%. However, dentists and dental students in L-LMICs showed significantly lower vaccine acceptance rates and trust in COVID-19 vaccines compared to their counterparts in UM-HICs. Our results provide important information to policymakers, highlighting the need for implementation of country-specific vaccine promotion strategies, with special focus on L-LMICs.

## Figures and Tables

**Table 1:** Sample characteristics: Data are presented as *n* (%).

	L-LMICs			UM-HICs				Total
	Pakistan	Egypt	India	KSA	Malaysia	Brazil	Turkey	
**Sample Size**	430	103	234	342	162	164	92	1527
**Age**								
18–29 years	416 (97%)	24 (23%)	163 (70%)	232 (68%)	109 (67%)	107 (65%)	62 (67%)	1113 (73%)
30–49 years	14 (3%)	65 (63%)	65 (28%)	108 (32%)	50 (31%)	45 (27%)	29 (32%)	376 (25%)
≥50 years	0 (0%)	14 (14%)	6 (3%)	2 (1%)	3 (2%)	12 (7%)	1 (1%)	38 (2%)
**Sex**								
Male	51 (12%)	57 (55%)	48 (21%)	185 (54%)	50 (31%)	43 (26%)	43 (47%)	477 (31%)
Female	379 (88%)	46 (45%)	186 (79%)	157 (46%)	112 (69%)	121 (74%)	49 (53%)	1050 (69%)
**Nationality**								
Native	428 (100%)	98 (95%)	233 (100%)	221 (65%)	160 (99%)	159 (97%)	78 (85%)	1377 (90%)
Foreigner	2 (0%)	5 (5%)	1 (0%)	121 (35%)	2 (1%)	5 (3%)	14 (15%)	150 (10%)
**Profession**								
Dentist	147 (34%)	100 (97%)	138 (59%)	191 (56%)	119 (73%)	102 (62%)	53 (58%)	850 (56%)
Dental student	283 (66%)	3 (3%)	96 (41%)	151 (44%)	43 (27%)	62 (38%)	39 (42%)	677 (44%)
**Place of work**								
Public	375 (87%)	35 (34%)	13 (6%)	41 (12%)	153 (94%)	64 (39%)	73 (79%)	754 (49%)
Private	43 (10%)	35 (34%)	218 (93%)	285 (83%)	7 (4%)	77 (47%)	15 (16%)	680 (45%)
Both	12 (3%)	33 (32%)	3 (1%)	16 (5%)	2 (1%)	23 (14%)	4 (4%)	93 (6%)
**Comorbidity**								
No	415 (97%)	82 (80%)	210 (90%)	302 (88%)	155 (96%)	139 (85%)	87 (95%)	1390 (91%)
Yes	15 (3%)	21 (20%)	24 (10%)	40 (12%)	7 (4%)	25 (15%)	5 (5%)	137 (9%)
**Previous COVID-19 infection**								
No	377 (88%)	76 (74%)	207 (88%)	293 (86%)	159 (98%)	126 (77%)	81 (88%)	1319 (86%)
Yes	53 (12%)	27 (26%)	27 (12%)	49 (14%)	3 (2%)	38 (23%)	11 (12%)	208 (14%)
**Vaccinated against COVID-19**								
No	353 (82%)	97 (94%)	167 (71%)	323 (94%)	81 (50%)	60 (37%)	59 (64%)	1140 (75%)
Yes	77 (18%)	6 (6%)	67 (29%)	19 (6%)	81 (50%)	104 (63%)	33 (36%)	387 (25%)

**Table 2:** Comparison of responses to different survey questions between L-LMICs and UM-HICs– Data are presented as % (*n*).

L-LMICs	UM-HICs	Total	*p* *
**Trust in Vaccines**			
**Vaccines are necessary to overcome the COVID-19 pandemic and get back to normal life.**			
69.5% (533/767)	82.9% (630/760)	76.2% (1163/1527)	<0.001 *
**I trust COVID-19 vaccines of ONLY certain companies.**			
52% (399/767)	37.9% (288/760)	45% (687/1527)	<0.001 *
**I think that vaccines against COVID-19 have been produced in a hurry without following recommended clinical trials and approval guidelines.**			
40.8% (313/767)	27% (205/760)	33.9% (518/1527)	<0.001 *
**I think that the companies involved in the development of the COVID-19 vaccines are doing it to make money.**			
22.8% (175/767)	29.6% (225/760)	26.2% (400/1527)	0.003 *
**I think that COVID-19 vaccines may have side effects which may show immediately or later on in life.**			
52.7% (404/767)	42.4% (322/760)	47.5% (726/1527)	<0.001 *
**I think companies producing COVID-19 vaccines are open about disclosing information on the side effects of the vaccine.**			
32.6% (250/767)	44.1% (335/760)	38.3% (585/1527)	<0.001 *
**Trust in Authorities**			
**I am happy with the way the health authorities have been managing the COVID-19 pandemic so far.**			
60.2% (462/767)	64.5% (489/758)	62.4% (951/1525)	0.08
**I am happy with the health authorities’ efficient organization of the COVID-19 vaccination program.**			
65.4% (502/767)	69.6% (529/760)	67.5% (1031/1527)	0.08
**Agreement to Accept a Vaccine**			
**I support a mandatory vaccination program for COVID-19.**			
72.1% (553/767)	69.1% (524/758)	70.6% (1077/1525)	0.20
**I will get vaccinated with the COVID-19 vaccine.**			
66.6% (511/767)	78.5% (596/759)	72.5% (1107/1526)	<0.001 *
**I will wait for other people to take the COVID-19 vaccine, as I am afraid to take it myself.**			
35.9% (275/767)	16.3% (124/760)	26.1% (399/1527)	<0.001 *
**I will delay taking the COVID-19 vaccine, as I feel there are others who deserve it more than me.**			
50.8% (390/767)	31.2% (237/760)	41.1% (627/1527)	<0.001 *
**Getting myself vaccinated for COVID-19 is important because I can also protect people with a weaker immune system.**			
78% (598/767)	81.9% (620/757)	79.9% (1218/1524)	0.06
**I will take the COVID-19 vaccine only if it is free.**			
29.5% (200/678)	29.1% (165/567)	29.3% (365/1245)	0.88
**Compared to the first dose of the COVID-19 vaccine, I fear that the second dose may have more chances to induce adverse side effects.**			
31.7% (227/716)	26.2% (154/587)	29.2% (381/1303)	0.03 *

*: *p* was calculated based on chi-square test.

**Table 3:** Agreement among dentists/dental students in different countries to accept a COVID-19 vaccine. Data are presented as %.

	Pakistan	Egypt	India	KSA	Malaysia	Brazil	Turkey	Total
**Total**	69.8%	33%	75.6%	62.5%	97.5%	96.3%	72.8%	72.5%
**Age**								
18–29 years	69%	29.2%	71.2%	62.5%	98.2%	96.3%	66.1%	72.4%
30–49 years	92.9%	32.3%	86.2%	61.7%	96%	95.6%	86.2%	72.5%
≥ 50 years	0%	42.9%	83.3% *	100%	100%	100%	100%*	76.3%
**Sex**								
Male	80.4%	40.4%	81.3%	64.1%	98%	97.7%	81.4%	72.9%
Female	68.3%	23.9%	74.2%	60.5%	97.3%	95.9%	65.3%	72.4%
**Profession**								
Dentist	70.1%	33%	81.2%	65.3%	97.5%	97.1%	84.9%	74.4%
Dental student	69.6%	33.3%	67.7% **	58.9%	97.7%	95.2%	56.4% **	70.2%
**Comorbidity**								
No	70.4%	32.9%	75.7%	62.9%	97.4%	97.1%	72.4%	73.2%
Yes	53.3%	33.3%	75%	59%	100%	92%	80%	66.2%
**Previous COVID-19 infection**								
No	70.3%	30.3%	74.9%	63%	97.5%	96%	71.6%	72.9%
Yes	66%	40.7%	81.5%	59.2%	100%	97.4%	81.8%	70.2%
**Updating self on the development of COVID-19 vaccines**								
No	51.4%	27.8%	52.9%	50%	93.3%	90.9%	62.5%	55.4%
Yes	75.7% **	34.1%	79.5% **	65.6% **	98%	96.7%	88.9% **	76.9% **
**Opinion about COVID-19 severity**								
Mild	85.7%	0%	71.4%	27.3%	100%	0%	20%	52.6%
Moderate	62%	28.6%	72.4%	59.3%	100%	90.5%	68.4%	64.9%
Severe	73.3%	37.9%	80%	68.4% *	96.7%	97.2%	81.6% *	78.2% *
**Compliance with COVID-19 preventive guidelines**								
Good	71.3%	32.3%	86.3%	66.1%	97.7%	98.9%	78.1%	76.1%
Moderate	68.4%	39.4%	69.4%	55.6%	96.7%	91.2%	61.5%	68.2%
Poor	72.2%	0%	40% *	33.3%	100%	100%	0%	64.7% *
**Anxiety about contracting COVID-19**								
Low	54.7%	16.7%	72.7%	56.3%	100%	100%	47.1%	61.3%
Moderate	69.3%	33.8%	72.3%	63.1%	98.6%	95.5%	80.9%	70%
High	75.8% *	34.6%	83.1%	68.7%	96.1%	96.3%	75%	80.9% *
**Concerned about the side effects of the vaccines**								
No	81.3%	48.1%	85.6%	74.2%	98.9%	97.6%	80%	83.5%
Yes	57.1% **	27.6%	66.7% **	48.4% **	95.9%	92.5%	67.3%	60.4% **

*: significance (*p* < 0.05) according to chi square test for trend. **: significance (*p* < 0.05) according to chi square test.

**Table 4:** Agreement among dentists/dental students in L-LMICs and UM-HICs to accept a COVID-19 vaccine. Data are presented as % (*n*).

	L-LMICS	UM-HICs	Total	*p*-Value between the Groups of Countries
**Total**	66.6% (511/767)	78.5% (596/759)	72.5% (1107/1526)	<0.001 **
**Age**				
18–29 years	68% (410/603)	77.6% (396/510)	72.4% (806/1113)	<0.001 **
30–49 years	62.5% (90/144)	78.8% (182/231)	72.5% (272/375)	0.001 **
≥ 50 years	55% (11/20)	100% (18/18)	76.3% (29/38)	0.001 **
**Sex**				
Male	66% (103/156)	76.3% (244/320)	72.9% (347/476)	0.02 **
Female	66.8% (408/611)	80.2% (352/439)	72.4% (760/1050)	<0.001 **
**Profession**				
Dentist	64.4% (248/385)	82.8% (384/464)	74.4% (632/849)	<0.001 **
Dental student	68.8% (263/382)	71.9% (212/295) **	70.2% (475/677)	0.4
**Comorbidity**				
No	67.6% (478/707)	78.9% (539/683)	73.2% (1017/1390)	<0.001 **
Yes	55% (33/60) **	75% (57/76)	66.2% (90/136)	0.014 **
**Previous COVID-19 infection**				
No	67.1% (443/660)	78.7% (518/658)	72.9% (961/1318)	<0.001 **
Yes	63.6% (68/107)	77.2% (78/101)	70.2% (146/208)	0.03 **
**Updating self on the development of COVID-19 vaccines**				
No	49% (77/157)	62% (93/150)	55.4% (170/307)	0.02 **
Yes	71.1% (434/610) **	82.6% (503/609) **	76.9% (937/1219) **	<0.001 **
**Opinion about COVID-19 severity**				
Mild	64.7% (11/17)	42.9% (9/21)	52.6% (20/38)	0.18
Moderate	61.7% (192/311)	68.7% (184/268)	64.9% (376/579)	0.08
Severs	70.2% (308/439) *	85.7% (403/470) *	78.2% (711/909) *	<0.001 **
**Compliance with COVID-19 preventive guidelines**				
Good	68% (230/338)	81.3% (425/523)	76.1% (655/861)	<0.001 **
Moderate	66.3% (266/401)	71.7% (152/212)	68.2% (418/613)	0.18
Poor	53.6% (15/28)	78.3% (18/23) *	64.7% (33/51) *	0.07
**Anxiety about contracting COVID-19**				
Low	56.8% (46/81)	64.1% (84/131)	61.3% (130/212)	0.29
Moderate	64.5% (284/440)	76.9% (269/350)	70% (553/790)	<0.001 **
High	73.6% (181/246) *	87.4% (243/278) *	80.9% (424/524) *	<0.001 **
**Concerned about the side effects of the vaccines**				
No	80.2% (291/363)	86.3% (378/438)	83.5% (669/801)	0.02 **
Yes	54.5% (220/404) **	67.9% (218/321) **	60.4% (438/725) **	<0.001 **

All comparisons were conducted between different levels of predictors at each cell^*^: *p* < 0.05 based on chi-square test for trend. **: *p* < 0.05 based on chi-square test.

**Table 5:** Predictors of agreement to accept a COVID-19 vaccine.

	Odds Ratio (95% Odds Ratio)	*p*
**Age**		
18–29 years	Ref	
30–49 years	0.66 (0.46–0.95) *	0.02
≥50 years	0.87 (0.36–2.11)	0.76
**Sex**		
Male	Ref	
Female	0.91 (0.68–1.23)	0.55
**Profession**		
Dentist	Ref	
Dental student	0.73 (0.55–0.99) *	0.04
**Comorbidity**		
No	Ref	
Yes	0.54 (0.35–0.84) *	0.01
**Previous COVID-19 infection**		
No	Ref	
Yes	1.25 (0.87–1.79)	0.23
**Updating self on the development of COVID-19 vaccines**		
No	Ref	
Yes	2.6 (1.95–3.48) *	<0.001
**Opinion about COVID-19 severity**		
Mild	Ref	
Moderate	1.38 (0.67–2.84)	0.39
Severe	2.23 (1.07–4.64) *	0.03
**Compliance with COVID-19 preventive guidelines**		
Good	Ref	
Moderate	0.81 (0.62–1.05)	0.12
Poor	0.73 (0.37–1.42)	0.35
**Anxiety about contracting COVID-19**		
Low	Ref	
Moderate	1.85 (1.29–2.66) *	0.001
High	3.19 (2.09–4.86) *	<0.001
**Concerned about the side effects of the vaccines**		
No	Ref	
Yes	0.25 (0.19–0.32) *	<0.001
**Income of the Country**		
L-LMICs	Ref	
UM-HICs	1.71 (1.31–2.23) *	<0.001

*: significance (*p*) is less than 5%. Ref: reference group.

## Data Availability

The data presented in this study are available on request from the corresponding author. The data are not publicly due to certain restrictions.

## Supplementary Materials

The following supporting information can be downloaded at: https://www.mdpi.com/article/10.3390/vaccines10101614/s1, Table S1: Agreement to different survey questions.
