# Supplementary material for: Determinants of COVID-19 Vaccine Acceptance among Dental Professionals: A Multi-Country Survey
Source: Vaccines (Basel). 2022 Sep 26;10(10):1614. doi: 10.3390/vaccines10101614 (PMC9610472; doi:10.3390/vaccines10101614)
Supplement: Supplementary file 1 [file vaccines-10-01614-s001.zip › vaccines-1862312-supplementary.pdf]

vaccines

MDPI

Table S1. Agreement to different survey questions. Data is presented as *n* (%).

Pakistan	Egypt	India	L-LMICs	KSA	Malaysia	Brazil	Turkey	UM-HICs	Total
**Trust in Vaccines**									
**Vaccines are necessary to overcome the COVID-19 pandemic and get back to normal life.**									
75.1% (323)	44.7% (46)	70.1% (164)	69.5% (533)	70.8% (242)	98.1% (159)	97% (159)	76.1% (70)	82.9% (630)	76.2% (1163)
**I trust COVID-19 vaccines of ONLY certain companies.**									
55.3% (238)	32% (33)	54.7% (128)	52% (399)	40.6% (139)	45.1% (73)	21.3% (35)	44.6% (41)	37.9% (288)	45% (687)
**I think that vaccines against COVID-19 have been produced in a hurry without following recommended clinical trials and approval guidelines.**									
35.3% (152)	70.9% (73)	37.6% (88)	40.8% (313)	36.8% (126)	12.3% (20)	15.9% (26)	35.9% (33)	27% (205)	33.9% (518)
**I think that the companies involved in the development of the COVID-19 vaccines are doing it to make money.**									
17% (73)	67% (69)	14.1% (33)	22.8% (175)	37.1% (127)	20.4% (33)	14% (23)	45.7% (42)	29.6% (225)	26.2% (400)
**I think that COVID-19 vaccines may have side effects which may show immediately or later on in life.**									
47.7% (205)	73.8% (76)	52.6% (123)	52.7% (404)	45.6% (156)	45.7% (74)	24.4% (40)	56.5% (52)	42.4% (322)	47.5% (726)
**I think companies producing COVID-19 vaccines are open about disclosing information on the side effects of the vaccine.**									
33.7% (145)	22.3% (23)	35% (82)	32.6% (250)	38% (130)	51.9% (84)	59.8% (98)	25% (23)	44.1% (335)	38.3% (585)
**Trust in Authorities**									
**I am happy with the way the health authorities have been managing the COVID-19 pandemic so far.**									
56.3% (242)	32% (33)	79.9% (187)	60.2% (462)	92.4% (316)	71% (115)	5.5% (9)	54.4% (49)	64.5% (489)	62.4% (951)
**I am happy with the health authorities' efficient organization of the COVID-19 vaccination program.**									
67.9% (292)	26.2% (27)	78.2% (183)	65.4% (502)	92.1% (315)	84% (136)	10.4% (17)	66.3% (61)	69.6% (529)	67.5% (1031)
**Agreement to Accept a Vaccine**									
**I support a mandatory vaccination program for COVID-19.**									
80.2% (345)	32% (33)	74.8% (175)	72.1% (553)	58.8% (201)	91.4% (148)	77.4% (127)	53.3% (48)	69.1% (524)	70.6% (1077)
**I will get vaccinated with the COVID-19 vaccine.**									
69.8% (300)	33% (34)	75.6% (177)	66.6% (511)	62.5% (213)	97.5% (158)	96.3% (158)	72.8% (67)	78.5% (596)	72.5% (1107)
**I will wait for other people to take the COVID-19 vaccine, as I am afraid to take it myself.**									
39.5% (170)	37.9% (39)	28.2% (66)	35.9% (275)	28.1% (96)	4.9% (8)	1.8% (3)	18.5% (17)	16.3% (124)	26.1% (399)
**I will delay taking the COVID-19 vaccine, as I feel there are others who deserve it more than me.**									
56.7% (244)	47.6% (49)	41.5% (97)	50.8% (390)	50.9% (174)	12.3% (20)	7.9% (13)	32.6% (30)	31.2% (237)	41.1% (627)
**Getting myself vaccinated for COVID-19 is important because I can also protect people with a weaker immune system.**									
79.5% (342)	53.4% (55)	85.9% (201)	78% (598)	77.1% (262)	96.3% (156)	82.3% (135)	73.6% (67)	81.9% (620)	79.9% (1218)
**I will take the COVID-19 vaccine only if it is free.**									
30.9% (117)	23.7% (23)	29.7% (60)	29.5% (200)	28.4% (93)	22% (22)	39.2% (29)	31.8% (21)	29.1% (165)	29.3% (365)
**Compared to the first dose of the COVID-19 vaccine, I fear that the second dose may have more chances to induce adverse side effects.**									
31% (121)	34.7% (35)	31.6% (71)	31.7% (227)	28.4% (93)	37.3% (38)	8.9% (8)	22.4% (15)	26.2% (154)	29.2% (381)
